# *Campylobacter *spp., *Enterococcus *spp., *Escherichia coli*, *Salmonella *spp., *Yersinia *spp., and *Cryptosporidium *oocysts in semi-domesticated reindeer (*Rangifer tarandus tarandus*) in Northern Finland and Norway

**DOI:** 10.1186/1751-0147-48-7

**Published:** 2006-06-14

**Authors:** N Kemper, A Aschfalk, C Höller

**Affiliations:** 1 Institute of Animal Breeding and Husbandry, Christian-Albrechts-University, Kiel, Germany; 2 Senate Department for Health, Social Services and Consumer Protection, Berlin, Germany; 3 Bavarian Agency for Health and Food Safety, Oberschleiβheim, Germany

## Abstract

The specific aim of this study was to assess the faecal shedding of zoonotic enteropathogens by semi-domesticated reindeer (*Rangifer tarandus tarandus*) to deduce the potential risk to human health through modern reindeer herding. In total, 2,243 faecal samples of reindeer from northern regions of Finland and Norway were examined for potentially enteropathogenic bacteria (*Campylobacter *species, *Enterococcus *species, *Escherichia coli*, *Salmonella *species and *Yersinia *species) and parasites (*Cryptosporidium *species) in accordance with standard procedures. *Escherichia coli *were isolated in 94.7%, *Enterococcus *species in 92.9%, *Yersinia *species in 4.8% of the samples and *Campylobacter *species in one sample only (0.04%). Analysis for virulence factors in *E. coli *and *Yersinia *species revealed no pathogenic strains. Neither *Salmonella *species nor *Cryptosporidium *oocysts were detected. The public health risk due to reindeer husbandry concerning zoonotic diseases included in this study has to be considered as very low at present but a putative epidemiological threat may arise when herding conditions are changed with respect to intensification and crowding.

## Introduction

Zoonotic organisms such as viruses, bacteria or parasites can possess the potential to cause severe diseases both in humans and animals. Free-ranging animals with sporadic or indirect contact to domestic livestock and humans may serve as reservoirs or sentinels for diseases. Transmission of these pathogens can occur directly from a reservoir to the susceptible animal or human being, for example through direct contact with free-ranging animals including cervids [1]. Indirect transmission occurs via vectors, i.e. mosquitos, by contamination of the environment through faecal shedding [2] or by consumption of venison [3]. The epidemiological situation in free-ranging, semi-domesticated animals like reindeer (*Rangifer tarandus tarandus*), however, is difficult to assess, in contrast to domestic animals. Conclusions derived from studies which focus on domestic animal species in extensive husbandry systems can only be used with caution. But it can be assumed that pathogens are transmitted easily through close animal contact and lead to high animal losses as it is known from intensive husbandry systems. This is of special importance as the crowding of reindeer for winter feeding is becoming more and more common, particularly in southern parts of Northern Finland.

With regard to the occurrence of possibly zoonotic pathogens in reindeer, data is rare. In view of the zoonotic enteropathogens examined in this study, *Campylobacter *species, *Enterococcus *species, *Escherichia coli*, *Salmonella *species and *Yersinia *species are among the most important bacteria which cause severe enteric diseases. In detail, various *Campylobacter *species (*C. coli, C. jejuni *subspecies *jejuni, C. hyointestinalis, C. lari *and *C. upsaliensis*) were isolated from humans with gastro-enteritis [4,5]. *Campylobacter hyointestinalis *was detected in healthy Finnish reindeer [6]. In a recent study about *Campylobacter *species and other bacteria in wild cervids in Norway, no *Campylobacter *species were detected in wild reindeer [7]. *Enterococcus *species are known as pyogenic organisms and can cause hospital acquired infections, but are primarily natural inhabitants of the gastrointestinal tract of humans and animals. *Enterococcus *species in reindeer have not yet been described. The virulence and pathogenesis of the examined bacteria depends on different factors. In *E. coli*, for instance, the ability to cause severe disease in humans and animals is associated with the occurrence of several virulence factors such as shigatoxins. Therefore, the presence of shigatoxin genes can indicate the virulence of certain strains, also known as shigatoxin-producing *E. coli *(STEC). In 50 faecal samples from wild Norwegian reindeer, 42 were positive for *E. coli*, but no shigatoxin-producing strains were identified [7]. Like STEC, *Campylobacter *species, *Salmonella *species and *Yersinia *species are a problem in meat production with a high infection risk for humans who consume contaminated products. *Salmonella *species have been found to be associated with mortality in reindeer in Finland and Sweden [8]. Among 153 wild Norwegian reindeer, no positive samples for *Salmonella *species were found [7]. Out of the former eleven known species of *Yersinia*, *Y. pestis *as the cause of plague, *Y. pseudotuberculosis *and certain biotypes of *Y. enterocolitica *are of great significance for human health. In Europe, the *Y. enterocolitica *biotypes O:3, O:9 and O:5,27 in particular are associated with human gastro-enteritis [9]. A novel species, *Y. aleksiciae*, identified by using 16S rRNA gene sequence type analysis and lysine decarboxylase (LDC) and in this points different from members of *Y. kristensenii*, was described by *Sprague & Neubauer *[10].

No data is available on the occurrence of zoonotic protozoa in Northern European reindeer such as *Cryptosporidium *species as a causative agent for heavy diarrhoea in humans and animals.

## Materials and methods

### Faecal samples

In total, 2,243 faeces samples from healthy reindeer, adults and calves, of both genders were taken over eleven month (June 2001 – April 2002) from eight Finnish and Norwegian free-ranging and corralled reindeer herds, considering parameters such as the degree of intensity of herding, location and season. The origin of samples and further information are given in Table 1. Samples were taken off the ground or per rectum from slaughter animals, sent to the laboratory directly after collection and kept frozen (-4°C) until being processed further within one week.

**Table 1:** Origin of faecal samples from 2,243 reindeer in Finland (F) and Norway (N)

**faecal samples**	**age**	**origin**	**month**	**number**	**n**	**n**_**total**_
free-living reindeer	all	F – Näkkälä	June	147		2,243
			
	all	F – Lappi	August	222	1,579	
			
	one year	F – Lappi	October-January	800		
			
	one year	N – Karasjok	September	410		
			
	one month	F – Kaamanen	June	40		
			
fenced reindeer	all	F – Näkkälä	February	100	664	
			
	all	F – Sallivaara	April	100		
			
	all	F – Palojärvi	March	325		
			
	all	F – Kiiminki	March	99		

### Examination for Campylobacter species

The examination was carried out by inoculating 1 g faecal material into 9 ml Preston broth (Oxoid). After 24 hours incubation in a microaerophilic atmosphere (5% oxygen, 10% carbon dioxide, 3% hydrogen and 82% nitrogen) at 37°C, a loopful of the enriched suspension was plated on Preston agar (Oxoid, Wesel, Germany) and incubated for 48 hours under the above-mentioned conditions. *Campylobacter*-like colonies were analysed by Gram-staining, catalase and oxidase tests, and further biochemical reactions (ApiCampy, bioMérieux, Marcy-l'Etoile, France). For all bacteria species, positive controls (ATCC, Manassas, USA) were used to approve the sensitivity of the culture methods.

### Examination for Enterococcus species

For the selective enrichment of *Enterococcus *species, 1 g faecal material was diluted in 9 ml glucose-azide broth (Merck, Darmstadt, Germany) and incubated for 48 hours at 37°C. A loopful of broth was then spread both on kanamycin-aesculin-azide agar (Merck) and Slanetz and Bartley agar (Oxoid). After 48 hours at 37°C suspicious colonies were Gram-stained and their biochemical reactions were analysed further by catalase and oxidase tests.

### Examination for Escherichia coli

*Escherichia coli *was isolated by adding 1 g faeces to 9 ml Gram-negative broth (Becton & Dickinson, Franklin Lakes, USA). After 24 hours of incubation at 37°C a loopful of broth was then plated onto Endo-c agar (Merck) and incubated under the above-mentioned conditions for 24 h. Typical metallic shiny colonies were subcultured on blood agar (Oxoid), incubated for 24 hours at 37°C and tested for their biochemical reactions applying API 20E (bioMérieux). PCR was used to detect the occurrence of shigatoxin1 and 2 genes (*stx1*, *stx2*), the intimin gene (*eae*) and EHEC-haemolysin gene (*hly*_*EHEC*_) as indicators for the pathogenicity of the isolated strains. Primers were developed with help of the European Molecular Biological Library database and the oligo 6.0 software (Molecular Biology Insight, Cascade, USA) and produced commercially (Invitrogen, Paisley, UK).

### Examination for Salmonella species

For the selective enrichment of *Salmonella *species 1 g faeces was inoculated into 14 ml of tetrathionate broth (Merck) and incubated for 24 hours at 37°C as described by *Baird *[11]. One ml of this enriched broth was brought into tetrathionate broth the next day and incubated for another 24 hours at 37°C. This enrichment step was repeated one more time. On the fourth day, one loopful of the cultured medium was plated both on *Salmonella-Shigella *agar (Difco) and Leifson agar (Merck). After 24 hours of incubation at 37°C presumptive *Salmonella *species colonies were Gram-stained and tested by API 20E (bioMérieux).

### Examination for Yersinia species

Cultural examination of *Yersinia *species was performed by adding 1 g faeces into 9 ml of Gram-negative broth and incubating for 48 hours at 21°C. One loopful of broth was then plated on *Yersinia*-selective agar (Difco) and incubated for another 48 hours at 21°C. Colonies with the typical bull's-eye appearance were subcultured on blood agar and Gram-stained and biochemical tests were subsequently carried out by use of API 20E (bioMérieux) and Micronaut (Merlin, Bornheim-Hersel, Germany). To detect various *Yersinia*-genes, crucial for the pathogenicity of the detected strains, PCR was performed using primers to detect the genes encoding *16SrRNA*, *yadA *and *v-antigen*.

### Examination for Cryptosporidium oocysts

For the detection of *Cryptosporidium *oocysts, immuno-magnetic separation was applied using Dynabeads anti-*Cryptosporidium *(Dynal Biotech, Oslo, Norway). Twenty μl of the immuno-concentrate were used for a direct immuno-fluorescence test (medac, Wedel, Germany) [12].*Cryptosporidium parvum *oocysts from a calf (Iowa isolate, Waterborne, USA) served as the positive control. Using a fluorescence microscope at x400-x1000 magnification *Cryptosporidium *oocysts appear as 6–10 μm in size, round or oval in shape with bright green fluorescence.

For statistical analyses, the data was evaluated with the Statistica 5.0 software (StatSoft Inc., Tulsa, USA), following the instructions of *Trampisch & Windeler *[13]. For all analyses, differences were considered significant at *P *≤ 0.05.

## Results

In 2,224 (99.2%) out of the total number of 2,243 faecal samples, one or more of the examined bacteria species were isolated.

*Campylobacter *species, identified as *Campylobacter hyointestinalis*, was detected in one faeces sample only (0.04%). *Enterococcus *species were isolated in 2,084 (92.9%) samples. *Escherichia coli *were isolated in 2,123 (94.7%) samples. Only few of the isolated *E. coli*-strains possessed genes encoding *stx1*, *stx2*, *eae *and *hly*_*EHEC*_, as shown in Table 2.

**Table 2:** Occurrence of *E. coli *toxin genes in 2,123 isolated strains (prevalences in parentheses) (*Kemper et al*. 2004)

	***stx1***	***stx2***	***eae***	***hly***_***EHEC***_	***eae+hly***_***EHEC***_
**n**_***E*.*coli*- **_= **2,123**	3 (0.14%)	0	11 (0.52%)	21 (0.99%)	2 (0.09%)

There was no evidence of the occurrence of *Salmonella *species nor *Cryptosporidium *species. In total, 108 (4.8%) strains of *Yersinia *species were isolated, consisting of *Y. enterocolitica *biogroup 1A (n = 29), *Y. intermedia *(n = 2), *Y. kristensenii *(n = 72), *Y. mollaretii *(n = 3) and *Y*. *rhodei *(n = 2).

**Figure 1:**
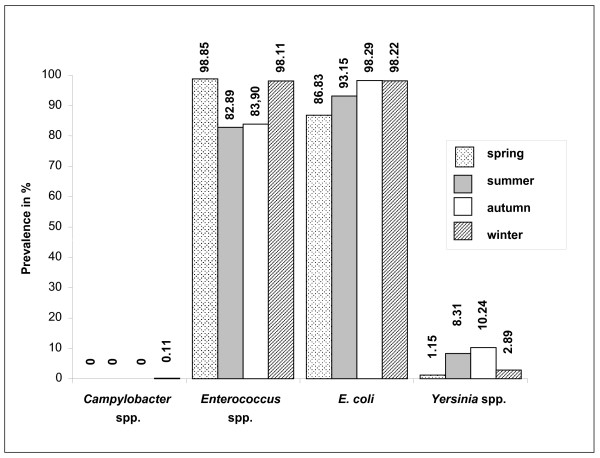
Prevalences of the examined pathogens in faecal samples taken in spring (n = 524), summer (n = 409), autumn (n = 410) and winter (n = 900).

No significant differences were found for *Enterococcus *species and *E. coli *with regard to the degree of intensity of reindeer herding, the season or the geographic origin, whereas the prevalence of *Yersinia *species differed significantly (p ≤ 0,001): prevalence for *Yersinia *species in free-ranging reindeer in summer and autumn were significantly higher than in fenced reindeer during winter, as shown in Figure 1. A direct comparison of fenced (n = 100) and free-living (n = 147) reindeer during different seasons was possible in Näkkälä, but with prevalences of 100,00% (fenced) and 65,99% (free-living) for *Enterococcus *species, 85,00% and 91,63% for *E. coli *and 1,00% and 0 % for *Yersinia *species no significant differences could be detected.

## Discussion

In reindeer, *Enterococcus *species and *E. coli *occurred at very high prevalence, showing the affiliation of these two species to the normal intestinal flora of healthy reindeer. With regard to *E. coli*, there are few reports of diseases caused by shigatoxin-producing bacteria in ruminants [14,15]. However, these bacteria are of extreme importance in causing severe diseases in humans [16]. As the genes encoding *stx1*, *eae *and *hly*_*EHEC *_were detected in very few of the isolated *E. coli*-strains, the human health risk due to *E. coli *excreted by reindeer can be considered very low at present. These results comply with two other studies detecting no *E. coli *O157:H7 in 1,387 faecal and 421 meat samples from reindeer [17] and no STEC in 50 faecal samples from reindeer [7]. It is known, however, that STEC virulence factors are mobile within bacterial populations [18]. Therefore, an increase in the occurrence of toxin genes in *E. coli *from reindeer cannot be excluded when influencing parameters such as herding conditions are changed.

*Yersinia *species were isolated in 108 samples. The identified species *Y. intermedia*,*Y. kristensenii*, *Y. mollaretii *and *Y*. *rhodei *had been isolated before from various environmental samples, food, healthy animals and healthy and diseased humans [19,20]. Even though these species are widely distributed in nature, their actual impact on human health is a matter of controversy. However, as they are isolated from persons with gastrointestinal disorders, the role of these species should not be disregarded [20]. These species seem to be well adapted to warm-blooded animals, including humans. After the identification of the novel species *Y. aleksiciae *[10], the isolates originally phenotyped as members of *Y. kristensenii *had to be investigated again (unpublished data). The isolated *Y. enterocolitica *strains belonged to biogroup 1A, which embraces the non-pathogenic European *Y. enterocolitica *strains, often isolated from environmental samples, foods, animal and human faeces [21]. *Yersinia *species, further analysed as *Yersinia pseudotuberculosis *serotype 1a, had also been isolated from reindeer before [22], but in samples from 35 Norwegian reindeer no *Yersinia *species were found [23]. The seasonal pattern of *Yersinia *species with an increase in the summer period can be attributed to the constant accidental incorporation of environmental strains which in this extent is not given in winter with limited survival conditions for bacteria.

*Campylobacter hyointestinalis *was isolated from one sample only. As the cultivation of *Campylobacter *species is difficult, the actual prevalence might be higher. *Campylobacter hyointestinalis *has hitherto been associated only sporadically with human gastrointestinal disorders [5,24]. Even though the prevalence of *Campylobacter *species in this study was very low, it shows that reindeer can be carriers. This is supported by a study from *Hänninen et al*. [6] who detected *Campylobacter hyointestinalis *at a prevalence of 6% in 399 healthy Finnish reindeer. *Lillehaug et al*. [7] isolated no *Campylobacter *species in 150 faecal samples of wild reindeer.

Neither *Salmonella *species nor *Cryptosporidium *oocysts were detected in reindeer in this study. Both pathogens had been isolated from the environment, farm animals and humans in Fennoscandia [25,26]. The occurrence of *Salmonella *species in Finnish reindeer is described by *Kuronen et al*. [8]. Regarding *Salmonella *species, the results of this study and of the study from *Lillehaug et al*. [7] with negative results for *Salmonella *species in 153 faecal samples from wild reindeer, indicate that wild cervids do not contract *Salmonella*-infections to any significant extent from other wildlife.

In *Rangifer tarandus*, a new genotype of *Cryptosporidium *closely related to *Cr. serpentis*, *Cr. muris *and *Cr. andersoni*, was isolated from 3 out of 49 caribou examined in Canada [27]. Concerning Northern European reindeer, no bibliographical references exist, but deriving from other species, the prevalence was expected to be higher in younger animals. This could not be supported by this study, identifying no *Cryptosporidium *oocysts at all.

All bacteria analysed in this study may be found in Northern Europe in the environment in aquatic, terrestrial and animal reservoirs [28] and have been isolated before from the intestinal tract of healthy or diseased ruminants world-wide [29,30]. Even though most of the isolated bacteria strains do not have the potential to cause severe human or animal health problems, certain strains might be a risk, especially for immuno-supressed, old or very young persons and animals. Therefore, one has to regard the epidemiological impact of transmission of these infectious agents from the environment to reindeer and man and *vice versa*, depending on a number of local factors. To recapitulate, the excretion risk of pathogens by reindeer has to be considered in the context of the extreme climatic conditions in the research area. Permafrost soils of the tundra and taiga are a domain of psychrophilic and psychrotolerant organisms [31], but as enteropathogens, living in intestines of warm-blooded animals at 37°C, are not adapted to these extreme northern environmental conditions, fast destruction is probable.

In conclusion, the enteropathogens examined were either not detected at all (*Salmonella *species and *Cryptosporidium *species), in very small numbers (*Campylobacter *species) or if detected, their virulence and pathogenicity was very low (*E. coli *and *Yersinia *species). The potential human and animal health risk from reindeer excreting various important enteropathogenic bacteria and *Cryptosporidium *species should be regarded very low at present. No differences could be found in the flora between fenced and free-living animals. However, especially if reindeer are crowded, e.g. for winter feeding, an increased prevalence of enteric pathogens excreted by reindeer and eventually an increased risk to the consumer has to be considered as is already known from other intensive animals husbandry systems worldwide.

## Conclusion

With respect to the investigated pathogens, the analysis of faecal samples from Norwegian and Finnish reindeer indicates that the animals do not represent an important source for zoonotic diseases at the moment. *Enterococcus *species and *E. coli *belong to the normal intestinal flora of reindeer. Climate conditions in the northern regions are a limiting factor to the survival of enteropathogens in the environment and might be a reason for the low prevalences of the other pathogens examined.

## Sammanfattning

*Campylobacter *spp., *Enterococcus *spp., *Escherichia coli*, *Salmonella *spp., *Yersinia *spp., og *Cryptosporidium *oocyster hos semi-domesticerade renar (*Rangifer tarandus tarandus*) i Finlands och Norges nordliga trakter

Målsättningen med studien var värdering av zoonotiska enteropatogeners fekala utbredning hos semi-domesticerade renar (*Rangifer tarandus tarandus*) för att bedöma potentialla risker för männsikans hälsa med modern renskötsel. Sammanlagt 2243 träckprover från renar i Finlands och Norges nordliga trakter har analyserats på potentiellt enteropatogena bakterier (*Campylobacter *species, *Enterococcus *species, *Escherichia coli*, *Salmonella *species och *Yersinia *species) och parasiter (*Cryptosporidium *species) med standardiserade metoder. *Escherichia coli *har isolerats i 94.7%, *Enterococcus *species i 92.9%, *Yersinia *species i 4.8% av proverna och *Campylobacter *species i bara ett prov (0.04%). Analys av virolensfaktorer i *E. coli *och *Yersinia *species visade inga patogena egenskaper. Varken *Salmonella *species eller *Cryptosporidium*-oocystor har påvisats. Den allmänna hälsorisken gällande zoonotiska sjukdomar på grund av renskötseln bedöms för tillfället som mycket låg men risken kan öka om renskötselns villkor ändras med hänsyn till intensitet och hållande i hägn.
